# Supplementary material for: Executive Function among Chilean Shellfish Divers: A Cross-Sectional Study Considering Working and Health Conditions in Artisanal Fishing
Source: Int J Environ Res Public Health. 2021 May 31;18(11):5923. doi: 10.3390/ijerph18115923 (PMC8199200; doi:10.3390/ijerph18115923)
Supplement: Supplementary file 1 [file ijerph-18-05923-s001.zip › Table S1.pdf]

Table S1: Decompression illness scores according to frequency and severity.

Severity	Frequency			Replacement	Total
	Never	Once in a lifetime	Several times	Medical diagnosis	
Mild symptoms [1] (x1)	0	1	2	3	
Neurological decompression sickness [2] (sum x2)	0	1	2	3	
Cerebral arterial gas embolism [3] (sum x3)	0	1	2	3	

[1] Specific symptoms of decompression sickness type 1 (in the former nomenclature) that is joint pain and skin alterations [12]. [2] Manifestations that could be more related to peripheral nerve and spinal cord involvement (regardless of their severity) [12]. [3] Considered when the diver had brain involvement after diving, with manifestations such as unconsciousness or seizures [12].

Example of application

A diver reported having suffered several episodes of mild decompression illness during his working life – non-diagnosed in a health center. In addition, he once experienced neurological decompression sickness, which was diagnosed in the local hospital. His history of decompression illness score is 8.

Severity	Frequency			Replacement	Total
	Never	Once in a lifetime	Several times	Medical diagnosis	
Mild symptoms (x1)	0	1	**2 (x1)**	3	**2**
Neurological decompression sickness (sum x2)	0	**1**	2	**3 (x2)**	**6**
Cerebral arterial gas embolism (sum x3)	**0 (x3)**	1	2	3	**0**
					**8**
