# Supplementary material for: Executive Function among Chilean Shellfish Divers: A Cross-Sectional Study Considering Working and Health Conditions in Artisanal Fishing
Source: Int J Environ Res Public Health. 2021 May 31;18(11):5923. doi: 10.3390/ijerph18115923 (PMC8199200; doi:10.3390/ijerph18115923)
Supplement: Supplementary file 1 [file ijerph-18-05923-s001.zip › Table S2.pdf]

Table S2: Additional test statistics of the linear regression models (Table 3)

1) **Percentage of perseverative responses**

Model Summary

Model	R	R Square	Adjusted R Square	Std. Error of the Estimate	Change Statistics				
					R Square Change	F Change	df1	df2	Sig. F Change
1	,420[a]	,177	,134	24,449	,177	4,159	5	97	,002

a. Predictors: (Constant), History of decompression illness score, dAge 40-49 years, Hazardous alcohol consumption, Depressive symptoms, dAge 50-59 years

ANOVA[a]

Model		Sum of Squares	df	Mean Square	F	Sig.
1	Regression	12429,274	5	2485,855	4,159	,002[b]
	Residual	57983,581	97	597,769		
	Total	70412,854	102			

a. Dependent Variable: Percentage of perseverative responses

b. Predictors: (Constant), History of decompression illness score, dAge 40-49 years, Hazardous alcohol consumption, Depressive symptoms, dAge 50-59 years

Coefficients[a]

Model		Unstandardized Coefficients		Standardized Coefficients	t	Sig.	95,0% Confidence Interval for B		Collinearity Statistics	
		B	Std. Error	Beta			Lower Bound	Upper Bound	Tolerance	VIF
1	(Constant)	13,972	5,422		2,577	,011	3,210	24,733		
	dAge 40-49 years	12,949	6,523	,240	1,985	,050	,004	25,895	,580	1,725
	dAge 50-59 years	14,749	6,755	,274	2,183	,031	1,341	28,156	,541	1,850
	Depressive symptoms	11,184	8,746	,121	1,279	,204	-6,174	28,542	,951	1,051
	Hazardous alcohol consumption	3,450	5,168	,063	,668	,506	-6,807	13,708	,969	1,033
	History of decompression illness score	1,252	,529	,235	2,368	,020	,202	2,301	,858	1,165

a. Dependent Variable: Percentage of perseverative responses

2) Percentage of perseverative errors

Model Summary

Model	R	R Square	Adjusted R Square	Std. Error of the Estimate	Change Statistics: R Square Change	F Change	df1	df2	Sig. F Change
1	,418[a]	,174	,132	17,785	,174	4,099	5	97	,002

a. Predictors: (Constant), History of decompression illness score, dAge 40-49 years, Hazardous alcohol consumption, Depressive symptoms, dAge 50-59 years

ANOVA[a]

Model		Sum of Squares	df	Mean Square	F	Sig.
1	Regression	6482,040	5	1296,408	4,099	,002[b]
	Residual	30681,300	97	316,302		
	Total	37163,340	102			

a. Dependent Variable: Percentage of perseverative errors

b. Predictors: (Constant), History of decompression illness score, dAge 40-49 years, Hazardous alcohol consumption, Depressive symptoms, dAge 50-59 years

Coefficients[a]

Model		Unstandardized Coefficients		Standardized Coefficients	t	Sig.	95,0% Confidence Interval for B		Collinearity Statistics	
		B	Std. Error	Beta			Lower Bound	Upper Bound	Tolerance	VIF
1	(Constant)	13,182	3,944		3,342	,001	5,354	21,010		
	dAge 40-49 years	10,057	4,745	,257	2,120	,037	,640	19,474	,580	1,725
	dAge 50-59 years	10,974	4,914	,280	2,233	,028	1,221	20,727	,541	1,850
	Depressive symptoms	7,736	6,362	,115	1,216	,227	-4,890	20,363	,951	1,051
	Hazardous alcohol consumption	2,430	3,759	,061	,646	,520	-5,032	9,891	,969	1,033
	History of decompression illness score	,881	,385	,228	2,290	,024	,118	1,644	,858	1,165

a. Dependent Variable: Percentage of perseverative errors

3) Percentage of non-perseverative errors

Model Summary

Model	R	R Square	Adjusted R Square	Std. Error of the Estimate	Change Statistics				
					R Square Change	F Change	df1	df2	Sig. F Change
1	,248[a]	,061	,013	12,728	,061	1,269	5	97	,284

a. Predictors: (Constant), History of decompression illness score, dAge 40-49 years, Hazardous alcohol consumption, Depressive symptoms, dAge 50-59 years

ANOVA[a]

Model		Sum of Squares	df	Mean Square	F	Sig.
1	Regression	1027,906	5	205,581	1,269	,284[b]
	Residual	15713,889	97	161,999		
	Total	16741,795	102			

a. Dependent Variable: Percentage of non-perseverative errors

b. Predictors: (Constant), History of decompression illness score, dAge 40-49 years, Hazardous alcohol consumption, Depressive symptoms, dAge 50-59 years

Coefficients[a]

Model		Unstandardized Coefficients		Standardized Coefficients	t	Sig.	95,0% Confidence Interval for B		Collinearity Statistics	
		B	Std. Error	Beta			Lower Bound	Upper Bound	Tolerance	VIF
1	(Constant)	24,821	2,823		8,793	,000	19,218	30,423		
	dAge 40-49 years	,014	3,396	,001	,004	,997	-6,725	6,754	,580	1,725
	dAge 50-59 years	3,621	3,517	,138	1,030	,306	-3,358	10,601	,541	1,850
	Depressive symptoms	-6,672	4,553	-,148	-1,466	,146	-15,709	2,364	,951	1,051
	Hazardous alcohol consumption	-,077	2,691	-,003	-,029	,977	-5,417	5,262	,969	1,033
	History of decompression illness score	-,458	,275	-,177	-1,664	,099	-1,004	,088	,858	1,165

a. Dependent Variable: Percentage of non-perseverative errors
