# Supplementary material for: Executive Function among Chilean Shellfish Divers: A Cross-Sectional Study Considering Working and Health Conditions in Artisanal Fishing
Source: Int J Environ Res Public Health. 2021 May 31;18(11):5923. doi: 10.3390/ijerph18115923 (PMC8199200; doi:10.3390/ijerph18115923)
Supplement: Supplementary file 1 [file ijerph-18-05923-s001.zip › Table S3.pdf]

Table S3: Sensitivity analysis of the linear regression models to assess the severity and frequency of decompression illness as a risk factor for reduced executive functioning among divers using severity and frequency of decompression illness as categorical variable. The models were adjusted for age, depressive symptoms, and hazardous alcohol consumption; N=103 divers with complete data.

	% Perseverative responses		% Perseverative errors		% Non-perseverative errors	
Adjusted R^2 (p_{Anova})	**0. 155 (p=0.001)**		**0. 156 (p=0.001)**		**0.007 (p=0.364)**	
	**B**	**95% CI**	**B**	**95% CI**	**B**	**95% CI**
**Age (years)**						
<40	0		0		0	
40-49	13.85	-0.13 – 27.83	10.68	0.53 – 20.83	0.95	-6.44 – 8.33
50-59	13.64	-0.53 – 27.81	10.11	-0.18 – 20.40	4.89	-2.60 – 12.38
**Depressive symptoms** [1] yes vs. no	11.51	-5.59 – 28.60	7.88	-4.54 – 20.30	-7.04	-16.07– 2.00
**Hazardous alcohol consumption** [2] yes vs. no	4.67	-5.59 – 14.93	3.31	-4.14 – 10.76	-0.08	-5.50 – 5.34
**History of decompression illness score** [3, 4] N (%)						
**0-1** 33 (32.0)	0		0		0	
**2** 23 (22.3)	-2.05	-16.66 – 12.57	-1.58	-12.19 – 9.04	-2.73	-10.46 – 4.99
**3 – 8** 22 (21.4)	13.96	-0.34 – 28.26	10.53	0.15 – 20.92	-6.21	-13.77 – 1.35
**≥ 9** 25 (24.3)	16.20	1.98 – 30.42	11.38	1.05 – 21.70	-6.82	-14.34 – 0.70

[1] Anhedonia or low mood "more than half the days" in the last two weeks. [2] AUDIT-C questionnaire score ≥5. [3] Score (in categories) of self-reported history of decompression illness according to severity and frequency of symptoms. Missing n= 1. [4] Comparison of medians of WCST (Kruskal-Wallis Test), median (min – max): (1) Percentages of perseverative responses: p <0.01, score 0 – 1= 18 (3 – 88), score 2= 20 (12 – 98), score 3 – 8= 29.5 (8 – 98), score ≥ 9= 32 (8 – 98). (2) Percentage of perseverative errors: p <0.01, score 0 – 1= 16 (3 – 66), score 2= 18 (12 – 73), score 3 – 8= 26.5 (7 – 73), score ≥ 9= 26 (8 – 73). (3) Percentage of non-perseverative errors: p= 0.18, score 0 – 1= 26 (4 – 66), score 2= 25 (2 – 38), score 3 – 8= 18.4 (2 – 59), score ≥ 9= 20 (2 – 58).
