# Supplementary material for: Executive Function among Chilean Shellfish Divers: A Cross-Sectional Study Considering Working and Health Conditions in Artisanal Fishing
Source: Int J Environ Res Public Health. 2021 May 31;18(11):5923. doi: 10.3390/ijerph18115923 (PMC8199200; doi:10.3390/ijerph18115923)
Supplement: Supplementary file 1 [file ijerph-18-05923-s001.zip › Table S4.pdf]

Table S4: Sensitivity analysis of the linear regression models to assess the severity and frequency of decompression illness as a risk factor for reduced executive functioning including fishermen. The models were adjusted for age, depressive symptoms, and hazardous alcohol consumption; N= 161.

	% Perseverative responses		% Perseverative errors		% Non-perseverative errors	
Adjusted R^2 (p_{Anova})	**0.140 (p=0.000)**		**0.138 (p=0.000)**		**0.003 (p=0.360)**	
	**B**	**95% CI**	**B**	**95% CI**	**B**	**95% CI**
**Age (years)**						
<40	0		0		0	
40-49	12.30	2.74 – 21.86	9.48	2.49 – 16.47	0.18	-4.75– 5.12
50-59	14.03	4.11 – 23.95	10.55	3.29 – 17.80	2.21	-2.91 – 7.33
**Depressive symptoms** [1] yes vs. no	15.74	4.88 – 26.59	11.35	3.41 – 19.29	-5.15	-10.76 – 0.45
**Hazardous alcohol consumption** [2] yes vs. no	6.57	-1.26 – 14.41	4.83	-0.90 – 10.55	-1.74	-5.78– 2.30
**History of decompression illness score** [3]	0.86	0.02 – 1.70	0.58	0.04 – 1.19	-0.23	-0.67 – 0.20

[1] Anhedonia or low mood "more than half the days" in the last two weeks. [2] AUDIT-C questionnaire score ≥5. [3] Score of self-reported history of decompression illness according to severity and frequency of symptoms. Missing n= 1.
