# Supplementary material for: Executive Function among Chilean Shellfish Divers: A Cross-Sectional Study Considering Working and Health Conditions in Artisanal Fishing
Source: Int J Environ Res Public Health. 2021 May 31;18(11):5923. doi: 10.3390/ijerph18115923 (PMC8199200; doi:10.3390/ijerph18115923)
Supplement: Supplementary file 1 [file ijerph-18-05923-s001.zip › Table S5.pdf]

Table S5: Linear regression models to assess the severity and frequency of decompression illness as a risk factor for reduced executive functioning among divers. The models were adjusted for age, educational level, and depressive symptoms; N=103 divers with complete data.

	**% Perseverative responses**		**% Perseverative errors**		**% Non-perseverative errors**	
Adjusted R^2 (p)	**0.253 (p=0.000)**		**0.237 (p=0.000)**		**0.032 (p=0.166)**	
	**B**	**95% CI**	**B**	**95% CI**	**B**	**95% CI**
**Age (years)**						
<40	0		0		0	
40-49	9.58	-2.42 – 21.58	7.74	-1.07 – 16.55	0.72	-5.95 – 7.38
50-59	10.29	-2.29 – 22.86	7.87	-1.36 – 17.10	4.17	-2.81 – 11.14
**Educational level**						
Complete secondary & superior	0		0		0	
Complete primary & incomplete secondary	1.36	-8.91 – 11.62	1.08	-6.46 – 8.61	1.48	-4.21 – 7.18
Incomplete primary	23.74	11.34 – 36.15	16.39	7.28 – 25.49	-4.26	-11.15 – 2.63
**Depressive symptoms** [1] yes vs. no	9.42	-6.75 – 25.58	6.53	-5.34 – 18.40	-6.17	-15.15 – 2.80
**History of decompression illness score** [2]	1.10	0.13 – 2.07	0.78	0.06 – 1.49	-0.41	-0.95 – 0.13

[1] Anhedonia or low mood "more than half the days" in the last two weeks. [2] Score of self-reported history of decompression illness according to severity and frequency of symptoms. Missing n= 1.
